# Does Smartphone Use Improve the Dietary Diversity of Rural Residents? Evidence from Household Survey Data from 5 Provinces

**DOI:** 10.3390/ijerph191711129

**Published:** 2022-09-05

**Authors:** Ting Jin, Lei Li

**Affiliations:** 1 College of Economics and Management, China Agricultural University, Beijing 100083, China; 2 Beijing Food Safety Policy and Strategy Research Base, China Agricultural University, Beijing 100083, China; 3 College of Economics and Management, Zhejiang A & F University, Hangzhou 311300, China; 4 Institute of Ecological Civilization, Zhejiang A & F University, Hangzhou 311300, China

**Keywords:** smartphones, dietary diversity, rural households, heterogeneity, transaction cost

## Abstract

The use of smartphones has profoundly changed the consumption patterns and living conditions of rural residents, but there is little research on how smartphone use affects the food consumption patterns of rural residents. This paper uses survey data from 1047 farmers from five Chinese provinces in 2020 to investigate the impact of smartphone use on the dietary diversity of rural residents, the underlying mechanism, and the corresponding group-level heterogeneity. The study finds that smartphone use has a significantly positive effect on the dietary diversity of rural residents and that the dietary diversity scores of rural residents who use smartphones to access the internet are a significant 4.2% higher than those of rural residents who do not. The results are robust to the use of instrumental variables and propensity score matching to account for potential endogeneity. The income effect and the transaction cost effect are the two mechanisms by which smartphone use improves the dietary diversity of rural residents. Compared with elderly residents and members of low-income households, young and middle-aged people and members of high-income households are more likely to use smartphones to improve their dietary diversity. The following recommendations for further improving the dietary diversity of rural residents are made: continue to increase the internet penetration rate and smartphone coverage rate in rural areas, conduct public welfare lectures on smartphone usage and nutrition and health knowledge, and improve the e-commerce distribution infrastructure in rural areas.

## 1. Introduction

Since the reform and opening up, with the rapid economic and social development, rural Chinese residents have bid farewell to the past model of a single-food diet and to subsistence-level self-sufficiency and are constantly shifting towards a market-oriented model of consuming diverse, nutritious, healthy foods. In recent years, the dietary diversity of rural Chinese residents has improved significantly, and the demand for fruits, vegetables, meat, poultry, fish, eggs, and milk has continued to increase. Farmers’ intake of energy, protein, and fat is increasing, and the sources of that energy, protein, and fat are also changing from plant-based foods to both animal- and plant-based foods. The traditional practice of providing a self-sufficient food supply is also increasingly shifting toward obtaining commercialized food through market-based channels in rural China. With the development of the market and the increment in income, the consumption of self-sufficient foods, such as grains, tends to decrease, while the consumption of high-protein foods that are sourced from market, such as meat and aquatic products, begins to rise [1,2].

When discussing achievements in rural China, we cannot ignore the diversity in the food consumption of rural residents. During China’s two sessions meeting in March 2022, it was emphasized that “it is necessary to establish a big food concept, start from better meeting the people’s needs for a better life, grasp the changing trend of the people’s food structure, and ensure the effective supply of meat, vegetables, fruits, aquatic products and other kinds of food while ensuring the food supply. Nothing can be done without it”. Improving dietary diversity is an inevitable requirement for practicing the concept of big food. Dietary diversity is fundamental for achieving a balanced diet, which can reduce the incidence of overweight, obesity, and other nutritional health problems [3]. Different foods contain different dietary components and nutrients, and the intake of different foods helps to meet the body’s diverse needs for energy and various nutrients. According to the “Healthy China 2019–2030 Action Plan”, the daily diet of the general population should include cereals, potatoes, vegetables and fruits, livestock, poultry, fish, eggs, milk, soybeans, nuts, and other foods. On average, residents should consume more than 12 types of food per day and more than 25 types per week. However, the unreasonable single-food dietary structure is still a large problem in many rural areas in China, especially in poor areas. The nutrient intake of rural residents is mainly sourced from common foods, such as grains, vegetables, and meat (livestock and poultry meat), and the consumption quantities of eggs, milk, fruits, and aquatic products are relatively small [4,5]. Food diversity among poor residents, elderly individuals, children, and members of other groups deserves special attention. The transportation in many rural areas is inconvenient and communication is poor, which hinders the ability to bring in outside information and food, resulting in a lack of nutritional knowledge and limited dietary diversity among farmers. There is still a large gap in the diversity of food consumption between rural residents and urban residents [6]. In this context, determining how to improve the dietary health of rural residents has become an important part of the “Rational Diet Action” included in the “Healthy China 2019–2030 Action Plan”.

In recent years, the internet revolution has not only profoundly affected the food consumption patterns and the composition of the food consumed by urban residents but has also reshaped the food consumption of rural residents in terms of food types and consumption patterns. According to the 47th Statistical Report on China’s Internet Development Status issued by the China Internet Network Information Center, as of December 2020, the percentage of internet users who use mobile phones to access the internet had reached 99.7%, and the number of individuals using mobile phones for online shopping had reached 781 million, of which rural internet users accounted for 309 million. The widespread popularity of smartphones has allowed many rural residents, who had not previously accessed the internet, to skip computers and become mobile internet users. In addition, e-commerce giants and platforms such as Taobao, JD.com, and Pinduoduo are accelerating along the “last mile” of e-commerce, making online shopping a normal part of life for farmers. In the past, the majority of rural residents ate mainly foods provided by themselves or obtained locally and purchased foods from other places through markets and shops. Today, an increasing number of rural residents are directly and deeply involved in the national food market and information platforms with the help of the power of the internet. They can search for and browse diverse food nutrition and health information without leaving home, buy foods online from all over the country, or sell agricultural products directly to consumers in all parts of the country. According to the “2016 China Rural E-commerce Consumption Trend Report” released by JD.com and the 21st Century Economic Research Institute, the percentage of mobile orders in rural areas is five percentage points higher than that in first-tier cities, and food and beverage orders are the fourth most frequently ordered products, following agricultural materials, maternal and child products, and personal care and cosmetics. In 2019, national online retail sales of agricultural products totalled 416.86 billion yuan, of which 71.8% were concentrated in three categories of products: fresh products, grain and oil condiments, and snack foods. It is worth noting that the income and age gaps within rural areas may prevent the internet from closing the rural–urban food and nutrition gap. If a large number of rural residents (especially low-income and elderly residents) do not enjoy the conveniences brought about by the digital transformation due to gaps in income and knowledge, the food and nutrition inequalities that already exist in rural areas may be exacerbated. This raises the questions that this research addresses: Has the widespread use of smartphones in rural areas changed the dietary health of rural residents, and if so, how? Does the impact of smartphones on food consumption diversity vary by income and age?

With the gradual growth in the popularity of mobile phones in rural areas, an increasing number of scholars have studied the impact of internet use on income, poverty reduction, agricultural production, and market accessibility among farmers [7,8,9]. However, insufficient attention has been given to the path and mechanism by which internet use affects the dietary health of rural Chinese residents. Some international scholars have conducted research on how mobile phone use affects food diversity and have found that mobile phone use has a positive impact on the dietary diversity of farmers [10,11,12]. However, these studies mainly consider rural areas in Latin America and Africa. For farmers in those regions, mobile phones are used more as an information communication tool. There is still a lack of research in countries such as China where the application of the internet is richer. In rural China, mobile phones are no longer limited to information communication functions due to the booming online markets and e-commerce. In addition, most domestic economic research on food still focuses on the relationships between income, market availability, education, habits, the new rural cooperative medical system and other factors, and the food safety and diversity experienced by rural residents [1,2,5,6]. The few studies related to the internet have either analysed the willingness of urban residents to buy food online and the corresponding factors of influence or have analysed the impact of the internet on the overall consumption of rural residents; there is still a lack of targeted causal analyses of how the internet affects the dietary health of rural residents.

In light of this reality, this article uses survey data from 1047 farmer households across five provinces—Jiangsu, Hebei, Hubei, Shaanxi, and Guangxi—in 2020 to conduct a detailed investigation on the relationship between internet use and dietary health among rural residents, the underlying mechanism, and any group-level heterogeneity. This article examines two channels by which smartphone use could affect dietary health: the income effect and online shopping. Understanding the mechanism by which the dietary health of rural residents is affected provides a realistic basis for guiding internet use to improve the food consumption of rural residents. The remainder of the paper is arranged as follows: the second part contains the theoretical hypothesis, the third part describes the research design, the fourth part presents the empirical analysis and results, the fifth part provides a further discussion, and finally, the conclusion and policy recommendations are provided.

## 2. Theoretical Hypothesis

Smartphone use is spreading rapidly in the rural areas of China, and online shopping has also penetrated further and is more convenient than that found in the rural areas of most other countries. Given the power of online shopping and e-commerce, how does smartphone use affect the food consumption behaviour of farmers? In the Chinese context, the impact of smartphone on the diversity of the food consumed among farmers and the underlying mechanisms may differ from those in other contexts. In what follows, two mechanisms underlying the effect of smartphone use on the food diversity of rural residents are analysed. These mechanisms are the income effect and the transaction cost effect.

### 2.1. Income Effect

Smartphone use can affect the diversity of farmers’ diets by increasing their incomes. Since smartphone use affects agricultural production and agricultural product markets, increased incomes may indirectly impact farmers’ food consumption behaviour. Research on the fishery market and fishers’ welfare in South India has found that mobile phone use can help fishers find more buyers in the market, practice arbitrage, and reduce the economic losses caused by unsold fresh fish and, thus, can improve fishers’ profits [13]. Phones can also help cushion income shocks. A stable income is essential for a high-quality diet, especially for small farmers in rural areas [11]. With the large-scale migration of rural labour, family members use the internet to remit money to each other, enabling them to share income risks and reducing the impact of external shocks on food consumption [7]. The use of smartphones provides farmers with a wealth of information and can also affect the composition of crops that they plant, their yield, and their technology adoption. For example, some studies have found that the price of soybeans in areas connected to the internet has increased by 1–3%, soybean yields have increased by 19%, and, finally, the net profit of farmers has increased by 33% [14]. According to survey data on apple farms, information acquisition is the largest marginal contributor to the adoption of organic farming among apple farmers, and information acquisition increases the probability that farmers are willing to accept organic agriculture by approximately 35.9 percentage points [15]. The use of the internet can free people from traditional agricultural labour and help them to engage in relatively high-income jobs, such as those in the manufacturing and service industries. This is conducive to increasing farmers’ labour productivity and thus increasing their income [16].

Internet use can increase the convenience of obtaining agricultural production inputs, technology, and product market and price information, thus generating income effects, and higher incomes often lead to higher food expenditures and improved family diets [17]. According to Bennett’s law, with an increase in the income level of residents, the diets of the residents tend to diversify. The consumption of low-value foods, such as grains, tends to decrease, while the consumption of high-value foods, such as livestock and poultry products, dairy products, and fruits, tends to increase [18]. A study on the availability of food among farmers in the Sichuan Basin found that economic availability has a significant impact on the consumption of a variety of foods that are currently underconsumed by farmers, while purchasing availability and household production have a significant impact on the consumption of only some foods. With the increase in income brought about by the use of smartphones, rural residents have increasingly higher requirements for nutrient-rich foods, and the composition of their diet tends to be more diversified.

### 2.2. Transaction Cost Effect

Smartphone use has an impact on the dietary diversity of rural residents by reducing transaction costs. Transaction costs refers mainly to the search costs to obtain information and the transaction costs of purchasing food online. The internet influences the dietary decisions of consumers through its effects on search costs and online shopping. As an increasing amount of food is purchased from markets, market access and market development can affect the diversity of the food consumed by farmers. For example, a study of the responses to 395 questionnaires distributed to farmers in Jiangsu, Henan, and Sichuan found that market access was a significant factor affecting individual dietary diversity and nutritional health [4]. The continual promotion and popularization of the internet has resulted in changes to consumption patterns and improvements in consumption, including among rural residents. Online shopping has had a strong impact on traditional commodity exchanges and consumption methods. Consumers can quickly and inexpensively obtain abundant and effective consumption information. The scope of transactions is greatly expanded. Consumers can transcend national boundaries and are not subject to geographical and time constraints. Purchases of required items are not limited [19]. Most rural residents use the internet to buy commodities that they cannot easily buy during the course of their daily life. The greatest value that e-commerce platforms such as Taobao, JD.com, and Pinduoduo can provide farmers may not be the ability to buy inexpensive commodities but the ability to buy those products that are not easily obtained by having such goods delivered [20]. Compared with traditional purchasing channels, online shopping has a wider selection of foods and less expensive prices, which not only reduces the cost of exchanging information and making purchases for farmers but also enables them to optimize their food consumption decisions [12].

Many farmers live far from food markets; thus, the transaction costs of purchasing food are high, and malnutrition is relatively common. Smartphones can help improve the efficiency of daily tasks, especially when people who are geographically distant communicate with each other. The lower transaction costs associated with smartphone use may have a positive impact on the quantity and variety of food available. The internet enables rural residents to purchase foods that are not available locally and at the same time increases their purchase options and enhances the diversity of the foods they consume. Mobile phones and mobile money can also help farmers coordinate and engage in collective actions for regular food purchases [21,22]. Online shopping allows more frequent market transactions without increasing personal transportation costs. In particular, more frequent transactions may have a particularly positive impact on the consumption of fresh and perishable foods, which are very important for the supply of micronutrients. Smartphones also provide convenient access to various news services and sources of information, increasing people’s nutritional knowledge and awareness, which in turn can help improve eating habits [23,24].

**Figure 1:**
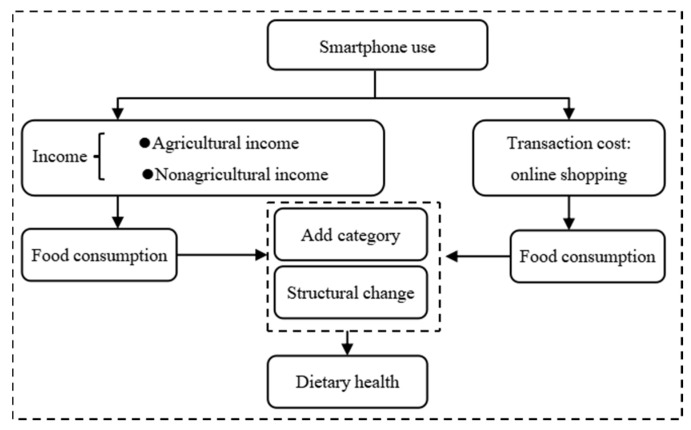
Theoretical Analytical Framework.

Based on the above analysis, this article argues that smartphone use can promote dietary diversity by increasing incomes and reducing transaction costs. The specific analytical framework is shown in Figure 1. Consequently, this paper proposes the following hypothesis:

Smartphone use improves the dietary health of rural residents.

## 3. Research Design

### 3.1. Data

The data used in this study come from the “Rural Chinese Production and Livelihoods in the New Era” project group of the China Agricultural University. The data were collected in 2020 for the eastern region (Jiangsu Province and Hebei Province), the central region (Hubei Province), and the western region (Guangxi Zhuang Autonomous Region and Shaanxi Province) through a home-based survey. The content of the questionnaire included basic household information and information on smartphone usage and food consumption. The survey used a multistage sampling method to determine which farmers to sample. In the first stage, two cities (counties) were selected from each province according to the per capita GDP and per capita disposable income of each province and city. In the second stage, in each sample district and county, the townships were categorized as high, medium, or low grade according to the level of per capita disposable income in recent years, and a township from each grade was selected for investigation. In the third stage, in each sampled township, the villages were divided into those with high levels of per capita disposable income in recent years and those with low levels, and one village from each level was randomly selected for investigation. In the fourth stage, in each sample village, the front station staff for the research group randomly selected households based on the villager roster, and the plan was to survey 15–25 households in each village. The age of the respondents was restricted to be within the range of 18–70 years old. The final sample size was 1047 households.

### 3.2. Variable Setting

(1) Explained variable. This paper explores the impact of smartphones on the dietary health of rural residents. The explained variable, dietary health levels, is measured with the dietary diversity scores of the farmers. The Household Dietary Diversity Score (*HDDS*) is an index for evaluating the quality of dietary nutrition proposed by the Food and Agriculture Organization of the United Nations (FAO) [25]. The calculation of the *HDDS* is based on FAO standards. Foods are divided into 12 categories: cereals, potatoes, vegetables, fruits, meat (including livestock and poultry), eggs, milk, fish and aquatic products, beans, fats, sugar, and miscellaneous (condiments, coffee, tea, etc.). According to FAO standards, 1 point will be given for each type of food consumed within 24 h or 7 days. Foods of the same type are not scored repeatedly, and the frequency and quantity of food consumption are not measured. The total score is obtained by summing the scores for the various foods together. The balanced diet pagoda from the “Dietary Guidelines for Chinese Residents (2016)” recommends “the daily intake of 8 types of foods such as grains and potatoes, soybeans and nuts, vegetables, fruits, eggs, aquatic products, milk, and livestock and poultry meat”. Similar to other Chinese studies, this research divided the different types of food into 8 categories: staple foods, meat, fruits, vegetables, milk, eggs, beans, and aquatic products. The *HDDS* was calculated based on the respondents’ diet review. Each food type consumed within 7 days scored 1 point, the same food was not scored repeatedly, and the total score could not exceed 8 points.

(2) Explanatory variables. The core explanatory variable in this article is smartphone usage. Whether the respondent uses a smartphone to surf the internet was selected as the explanatory variable and was treated as a binary indicator, with 1 representing yes and 0 representing no.

(3) Control variables. Individual characteristics, such as gender, age, education level, marital status, nutritional and health knowledge scores, status as the person who most frequently buys food at home, and status as the person who most frequently cooks food at home, were used as control variables. In addition, family characteristics, such as per capita annual income, the number of elderly people over 60 years old in the home, and the number of children aged 6–18 years old in the family, were included. Village characteristics, such as per capita annual income and distance from the village to the county seat, were also controlled for.

The descriptions and settings for specific variables are shown in Table 1.

Table 2 shows the percentage of farmers who do not consume certain types of food within a week. The results suggest that the dietary diversity of the interviewed farmers needs to be further improved, especially in terms of dairy and aquatic products. This is in line with the acknowledged gap between reality and the requirement in the “Healthy China 2019–2030 Action Plan” that “the daily diet of the general population should include cereals, potatoes, vegetables and fruits, livestock, poultry, fish, eggs, milk, soybeans, nuts and other foods, with an average daily intake of more than 12 kinds of food, and a weekly intake of 25 kinds or more”. The gap in the consumption of milk was the greatest; approximately 80% of the interviewed farmers do not drink milk often; the second largest gap was in the consumption of aquatic products, as nearly half of the interviewed farmers do not consume aquatic products on a daily basis. The vast majority of the interviewed farmers consume staple foods and vegetables on a daily basis, but there are also a small number of farmers who do not consume beans, fruits, or eggs within a week. In each of the different regions, drinking milk has not yet become a dietary habit among farmers, and the percentage of farmers who do not consume milk does not exhibit regional differences. The consumption of aquatic products occurs mainly in Jiangsu, Hubei, and Guangxi, which is related to the relatively well-developed local aquatic product industry. The percentage of farmers who do not consume beans weekly is the highest in Guangxi and the lowest in Jiangsu. This suggests that many rural residents do not yet choose their diets to ensure dietary balance and dietary diversity.

### 3.3. Model Setting

This paper analyses the impact of smartphones on the dietary health of rural residents, which is measured with the *HDDS*. The specific empirical model is as follows:

(1) $$HDDS_{i}=\alpha +\beta Mob_{i}+\gamma X_{i}+\mu_{c}+u_{i}$$

$HDDS_{i}$ represents the dietary diversity score for rural household *i*, $Mob_{i}$ indicates the usage of smartphones by the members of rural household *i*, $X_{i}$ represents the control variables, $\mu_{c}$ represents the regional fixed effects, and $u_{i}$ represents the error term.

The above baseline model may result in biased and inconsistent estimates due to endogeneity problems. The problem of endogeneity has two main sources. One is the omitted variable problem. Some unobservable factors, such as the dietary habits and consumption knowledge of rural residents, may affect dietary diversity, leading to problems such as omitted variables; the second is the problem of simultaneous cause and effect. That is, the ever-increasing consumer demand may drive rural residents to purchase and use smartphones, which creates the problem of simultaneous cause and effect. To address these problems, this article uses instrumental variables. This is explained in detail below.

## 4. Empirical Results and Analysis

### 4.1. Benchmark Regression

As shown in Table 3, column (1) includes only the core explanatory variables, column (2) adds other control variables, column (3) includes only the regional dummy variables, and column (4) adds other control variables to the specification in the third column. By comparing the results, we can see that the use of smartphones significantly improves the dietary diversity scores of rural residents. According to the results in column (4), compared with rural residents who do not use smartphones to access the internet, rural residents who do use smartphones have dietary diversity scores that are significantly higher by 4.2%. This validates the hypothesis proposed during the theoretical analysis that smartphone use increases the dietary diversity of farmers.

The results for the control variables are basically in line with theoretical expectations. Individual characteristics, such as education level, nutrition and health knowledge level, and per capita annual income, have a significantly positive impact on the dietary diversity scores of rural residents at the 1% level. This shows that improving education levels, nutrition and health knowledge, and per capita annual income can help improve the dietary diversity of rural residents. Regarding family characteristics, there is a positive relationship between per capita annual income and the dietary diversity of rural residents, which is consistent with the results predicted by food consumption theory. Among the village characteristics, the village-level per capita annual income has a significantly positive impact on dietary diversity scores at the 10% level. In addition, the distance from the village to the county seat has a significant negative impact on dietary diversity scores at the 1% level.

### 4.2. Instrumental Variable Method

To address possible endogeneity problems, this article has carefully selected control variables, controlling for individual characteristics, family characteristics, village characteristics, and regional fixed effects as much as possible to reduce the bias caused by unobservable factors. However, there may still be endogeneity problems. Furthermore, in this article, instrumental variables for the smartphone usage of rural residents are used to reduce the bias and inconsistency caused by possible endogeneity.

Effective instrumental variables must meet two conditions: one is that they must not be related to the random disturbance terms, and the other is that they must be related to the endogenous variables. In this paper, broadband coverage in the village and the number of years that broadband has been installed in the household are used as instrumental variables for individual smartphone usage. In terms of relevance, first, village-level broadband coverage captures the penetration of the internet into a region, and the behaviour of people living in the same village can easily be affected by their interactions with others [26]. Under normal circumstances, to reduce costs, network infrastructure is laid by region. If the network infrastructure is built in a given area, different farmers in the corresponding village can easily access the network at any time. Therefore, the interactions in expected behaviour will cause the internet usage of specific rural households in the village to be correlated with the average level of internet usage in the village. Therefore, the more developed the regional internet infrastructure is, the greater the probability of rural residents using smartphones to access the internet. Second, the longer a household has had broadband installed, the more likely it is for members of that household to use smartphones to surf the internet in order to maximize the utility of the broadband connection.

Regarding the exogeneity condition, first, whether network infrastructure has been laid in a certain area is greatly affected by factors such as the economic level of the area and road construction. Therefore, village-level broadband coverage may be related to factors such as the economic level of the village and the distance from the county seat and, thus, may affect the food consumption of the rural residents in the village. To address this issue, in this article, the economic status of the village and the distance from the county seat are controlled for so that the village-level broadband coverage is not related to the food consumption of rural residents at the micro (individual) level. In addition, the food consumption of rural residents cannot adversely affect village-level broadband coverage, so this instrument is not subject to the problem of reverse causality. In addition, broadband coverage in the village greatly reduces the measurement error problem that may exist in the data on the use of individual smartphones. The design of this instrumental variable has been adopted by other scholars [27,28]. Secondly, similar to village-level broadband coverage, the number of years that a household has had a broadband connection may be related to factors such as household income, education level, the number of elderly household members, and the number of children. There are more infants, more left-behind children, and more children overall in rural areas. To facilitate children’s education and their contact with their working parents, family broadband coverage may be widespread. For this reason, in this article, such variables are controlled for to ensure that household-level broadband coverage is not related to the food consumption of the rural household members. In addition, the number of years that broadband has been installed in the home also greatly reduces the measurement errors that may exist in the data on the use of individual smartphones. Therefore, logically, the village-level broadband coverage rate and the number of years that broadband has been installed in each home meet the relevance and exogeneity conditions for instrumental variables. Of course, this claim needs further testing. 

Table 4 shows that the *p* value for the Durbin–Wu–Hausman (DWH) test is 0.000, which means that the exogeneity hypothesis can be rejected at the 1% level, indicating that smartphone use is an endogenous variable. The Cragg–Donald Wald F statistic is 24.573, and the critical value is 19.93 when the error is greater than 10%, which eliminates concerns about weak instrumental variables [29]. The *p* value for the overidentification test (Hansen J statistic) is 0.079, indicating that the null hypothesis that all the instrumental variables are exogenous and cannot be rejected at 5% significant level. The above test results show that using village-level broadband coverage and the duration of home broadband installation as instrumental variables can be used to more accurately identify the impact of smartphone usage on the dietary diversity of rural residents.

In Table 4, column (1), the traditional two-stage least-squares (2SLS) method is used for estimation. In column (2), the limited information maximum likelihood (LIML) estimation method, which is more robust to weak instrumental variables, is used. In column (3), the generalized method of moments (GMM), which is more effective under heteroscedasticity conditions, is used for estimation, and in column (4), iterative GMM is used for estimation. Overall, the estimation results from the four methods all show that smartphone use has a significant positive impact at the 1% level on the dietary diversity of rural residents, and that the estimation coefficients are relatively close, further indicating that the estimation results are robust and credible.

### 4.3. Robustness Test

Although the instrumental variable method has been used to overcome, as much as possible, the endogeneity problems caused by omitted variables, reverse causality, etc., due to data and variable limitations, there may still be selection bias in the analysis. That is, whether it is possible to use a smartphone to surf the internet is not randomly assigned but is the result of rural residents’ self-selection. Because of this, direct regression is likely to lead to selection bias. To address this issue, this article uses propensity score matching to construct a counterfactual to correct this bias and further verify whether the positive effect of smartphone use on the dietary diversity of rural residents is consistent and stable. However, propensity score matching mainly controls for the influence of observable variables. If the observable variables are selected improperly or too few are used, estimation errors can easily occur. Therefore, this study uses propensity score matching as a robustness test only.

This paper calculates the average treatment effect (average treatment effect on the treated, ATT) between two groups of residents—those who use smartphones to surf the internet and those who do not—after matching. Table 5 shows the results using nearest neighbour matching, radius matching, kernel matching, and local linear regression matching. The ATT results all show that, after eliminating the systematic observable differences between subsamples, the use of smartphones to surf the internet has a significant positive impact on the dietary diversity of rural residents.

## 5. Further Discussion

### 5.1. Mechanism Analysis

This article explores whether the income and transaction cost effects are mechanisms by which the effect of smartphone use is transmitted to residents’ dietary diversity. To effectively identify the transmission mechanism, the following sequential recursive models are established to test whether income has a mediating effect. (1) As a test for the influence of smartphone use on the dietary diversity of rural residents, if the coefficient on smartphone use is significant, it shows that smartphone use has a significant impact on the dietary diversity of rural residents. (2) Then, the next step is to test the impact of smartphone use on income as an intermediary variable and whether online food is purchased. If the coefficient on smartphone use is significant, it indicates that smartphone use can affect the income of rural residents and their online food shopping behaviour. (3) Income variables are added to the model in step (1), and whether online food is purchased is tested. If the effect of the mediating variable is significant and the coefficient on smartphone use is smaller than or even insignificant relative to the coefficient obtained in step (1), then income and online food shopping have either partial or full mediating effects.

According to the test described above, the following empirical model is established:

The first step is to test whether the use of smartphones affects the dietary diversity of rural residents.

(2) $$Y_{i}=\alpha +\beta Mob_{i}+\gamma D_{i}+\mu_{c}+u_{i}$$

The second step is to check whether the use of smartphones affects income and online food shopping.

(3) $$I_{i}=\alpha +\beta Mob_{i}+\gamma D_{i}+\mu_{c}+u_{i}$$

The third step is to include the smartphone usage variable, the income variable, and the online shopping variable in the model at the same time.

(4) $$Y_{i}=\alpha +\beta Mob_{i}+\varphi I_{i}+\gamma D_{i}+\mu_{c}+u_{i}$$

$D_{i}$ represents the control variables. Aside from the inclusion of the resident’s annual income, it is the same as the set of control variables $X_{i}$ in Equation (1). $I_{i}$ represents the intermediary variables: resident income and whether the household buys food online. The results of the first step are shown in Table 6, the results of the second step are shown in Table 7, and the results of the third step are shown in Table 8.

The test results for the first step, presented in Table 6, show that the use of smartphones has a significant impact on the dietary diversity of rural residents. The test results for the second step, presented in Table 7, show that the use of smartphones has a significant impact on residents’ income and online shopping behaviour. Compared with those who do not use a smartphone to surf the internet, residents who do use a smartphone to surf the internet are more likely to shop online, and their annual income is also higher. The test results for the third step, presented in Table 8, show that the use of smartphones significantly affects the dietary diversity of rural residents and that income and online shopping also have significant effects on the dietary diversity of residents. In addition, when the intermediary variables are controlled for, the impact of smartphone use on residents’ dietary diversity (see model (4)) declines relative to the estimated coefficient on smartphone use in model (2) (see Table 6). Therefore, economic status as expressed by income and online shopping behaviour, i.e., the decision regarding whether to buy food online, can be considered to have an important mediating effect in the process by which smartphone use affects the dietary diversity of rural residents, indicating that smartphone use can be increased by relaxing budget constraints and that reducing transaction costs has a positive impact on the dietary diversity of rural residents. The above results verify the theoretical hypothesis.

### 5.2. Heterogeneity Analysis

The above discussion covers in detail the causal relationship between smartphone use and the dietary diversity of rural residents, but it does not answer the question of the heterogeneity in the impact of smartphone use among different groups. This Section examines the heterogeneous effects of smartphone use on the dietary diversity of rural residents according to different characteristics, such as age and income. Table 9 reports the estimated results.

Regarding the heterogeneity by age group, 60 years old is usually used to indicate whether an individual is elderly, so this article uses age 60 to define different age groups. Columns (1) and (2) of Table 9 show that, among people under 60 years old, smartphone use can significantly improve dietary diversity. Regarding the heterogeneity by income level, since the average annual income is 14,423.84 yuan, this article divides individuals into different income groups according to this standard. Columns (5) and (6) of Table 9 show that, among members of the higher-income group, smartphone use can significantly improve dietary diversity, but it has no effect among members of the low-income group. One possible reason is that, compared with low-income households, high-income households are more willing to use e-commerce platforms to buy food from all over the world. The above results show that the impact of smartphone use on the dietary diversity of rural residents varies significantly by age and economic condition. Smartphone use has a more significant impact on young and middle-aged individuals and high-income households than among elderly individuals and low-income households.

## 6. Conclusions

The “Healthy China 2030” Plan Outline, reviewed and approved in 2016, proposed “[focusing] on solving the problems of micronutrient deficiencies and excessive intake of high-calorie foods such as fats and oils for some people, and gradually [solving] the coexistence of nutritional deficiencies and excesses among residents”. The digital technology represented by the internet has set off a revolution in the understanding of food nutrition, ingredients, and purchase channels in rural China. Using survey data from five provinces in 2020, this article examines the impact of smartphone use on the dietary health of rural residents and explores the group-level heterogeneity in smartphone use and its impact mechanism. The results show that internet use has a significant positive effect on the dietary health of rural residents. Specifically:

(1) Compared with rural residents who do not use smartphones to access the internet, rural residents who do use smartphones to access the internet have food consumption diversity scores that are significantly higher by 4.2%. To take into account the possible endogeneity issues, this article selects appropriate instrumental variables to address endogeneity and uses propensity score matching; the results are still robust. (2) Further analysis of the impact mechanism shows that the use of smartphones improves the diversity of the foods in their diet by increasing the income level of rural residents and reducing transaction costs. That is, the relaxation of budget constraints and the increased efficiency of food acquisition are important mechanisms by which the use of smartphones improves the dietary diversity of rural residents. (3) The effect of smartphone use on the dietary diversity of rural residents is heterogeneous across groups. The analysis reveals that there are significant differences in the impact of smartphone use on the dietary diversity of rural residents of different ages and with different incomes. Smartphone use has a more significant impact on young people and high-income households than on elderly people and low-income households.

On the basis of the above analysis, this article makes the following suggestions for making full use of the internet to promote the dietary diversity of rural residents: (1) policymakers should continuously increase internet penetration and smartphone coverage in rural areas, as doing so will help narrow the urban-rural dietary health gap. In 2020, the internet penetration rate in rural areas was only 55.9%, and a large number of rural residents were still excluded from internet use due to an incomplete rural network infrastructure, a lack of skills, and limited education. Continuing to increase the internet penetration rate and smartphone coverage rate in rural areas will help consolidate poverty alleviation and rural revitalization and expand their effects, help ensure food safety, and reduce nutrition and health issues in rural areas. (2) To improve food consumption diversity in rural China, local governments could conduct lectures for public welfare, such as the use of smartphones, and provide knowledge about healthy eating patterns. In rural areas, nutritional vulnerable groups include, but are not limited to, elderly populations, young children, pregnant women, and low-income groups. These people have no resources or money to learn corresponding knowledge so as to improve the food consumption diversity. (3) To make rural residents better facilitate food consumption, the government needs to improve e-commerce distribution infrastructure in rural areas. In addition to the internet penetration rate, the main component of the external environment that affects whether rural residents can shop online is the construction of local village roads, express delivery outlets, and other related supporting facilities. To further expand the coverage of rural e-commerce, it is necessary to focus on solving the “last mile” problem and achieve full coverage of express delivery outlets in all administrative villages. A number of comprehensive commercial service centres and logistics distribution centres in county towns can be upgraded; a group of township commercial centres can be upgraded in townships; in villages, a group of new chain convenience stores can be built, thus increasing the rapid delivery and distribution of agricultural products according to local conditions.

It should be noted that the issues discussed in this article are related mainly to the difference between being online and being offline; this article does not examine the impact of the quality of smartphone use on the dietary diversity of rural residents, and this issue will be further investigated in the future. In fact, however, using equipment to obtain information or for shopping requires only the basic skill of identifying the value of information and quickly obtaining the required information. The ability to transform valuable information into nutrition and health behaviours is even more important. Especially when basic technologies become popularized over time, differences in the latter three capabilities will determine whether the internet becomes particularly important for the dietary diversity of residents.

## Figures and Tables

**Table 1:** Sample descriptive statistics.

Variable	Meaning/Value	Sample Size	Mean	Standard Deviation	Minimum	Maximum
Explained variable						
Household Dietary Diversity Score	Continuous value	1047	6.011	1.221	1	8
Explanatory variable						
Smartphone use	Yes = 1 No = 0	1047	0.637	0.441	0	1
Individual characteristics						
Gender	Male = 1 Female = 0	1047	0.518	0.499	0	1
Age	Years	1047	54.973	9.984	19	69
Education level	Years	1047	6.550	3.509	0	19
Marital status	Married = 1 Other = 0	1047	0.937	0.241	0	1
Nutrition and health knowledge score	Continuous value	1047	6.712	1.681	0	9
Respondent is the person who most frequently buys food at home	Yes = 1 No = 0	1047	0.651	0.477	0	1
Respondent is the person who most frequently cooks meals at home	Yes = 1 No = 0	1047	0.615	0.487	0	1
Family characteristics						
Per capita annual income (Add 1 to take the logarithm)		1047	9.092	1.380	0	11.513
Number of people over age 60 in the family		1047	0.462	0.617	0	3
Number of children aged 6–18 in the family		1047	0.564	0.870	0	5
Village characteristics						
Per capita annual income (Add 1 to take the logarithm)		1047	9.251	0.535	8.161	10.463
Distance from village to county seat	Miles	1047	50.479	42.167	0	150

**Table 2:** Percentage of farmers who do not consume certain types of food in one week (%).

Number	Category	Full Sample	Jiangsu	Hebei	Hubei	Shaanxi	Guangxi
1	Milk	78.01	75.00	68.88	81.64	79.12	84.56
2	Aquatic products	45.79	18.59	73.03	26.57	78.57	29.34
3	Beans	22.37	9.62	15.35	25.12	15.93	39.00
4	Fruit	21.61	23.72	17.01	32.85	27.47	11.58
5	Eggs	17.50	12.18	14.94	18.84	23.63	17.76
6	Meat	14.24	22.44	12.03	17.39	19.23	5.41
7	Vegetables	0.48	0.64	0.41	7.25	0.55	0.77
8	Staple foods	0.10	0.64	3.32	4.35	2.75	7.72

**Table 3:** Baseline model.

Explanatory Variables	Explained Variable: Household Dietary Diversity Score			
Core Explanatory Variables	(1)	(2)	(3)	(4)
Use smartphone to surf the internet	0.467 *** (0.081)	0.223 ** (0.089)	0.473 *** (0.081)	0.229 *** (0.088)
Individual characteristics				
Gender (male)		0.075 (0.098)		0.077 (0.098)
Age		−0.002 (0.005)		−0.002 (0.005)
Education level		0.090 *** (0.012)		0.089 *** (0.013)
Marital status		0.107 (0.160)		0.091 (0.160)
Nutrition and health knowledge score		0.082 *** (0.025)		0.083 *** (0.025)
Respondent is the person who most frequently buys food at home		0.079 (0.108)		0.080 (0.108)
Respondent is the person who most frequently cooks meals at home		−0.130 (0.119)		−0.127 (0.119)
Family characteristics				
Per capita annual income (Add 1 to take the logarithm)		0.079 *** (0.028)		0.086 *** (0.029)
Number of people over age 60 in the family		−0.081 (0.064)		−0.071 (0.064)
Number of children aged 6–18 in the family		0.010 (0.046)		0.013 (0.047)
Village characteristics				
Per capita annual income (Add 1 to take the logarithm)		0.093 (0.071)		0.125 * (0.073)
Distance from village to county seat		−0.003 *** (0.001)		−0.003 *** (0.001)
Regional fixed effects	N	N	Y	Y
Sample size	1051	1047	1050	1046
F statistic	33.310	13.360	6.89	10.150
R^2^	0.031	0.138	0.035	0.144

Note: *** indicates *p* < 0.01, ** indicates *p* < 0.05, and * indicates *p* < 0.10 here.

**Table 4:** Instrumental variable regressions.

Variable	2SLS (1)	LIML (2)	GMM (3)	Iterative GMM (4)
Smartphone use	1.482 *** (0.428)	1.572 *** (0.463)	1.511 *** (0.428)	1.510 *** (0.428)
Control variables	Y	Y	Y	Y
Regional fixed effects	Y	Y	Y	Y
DWH test *p* value	0.000			
Cragg–Donald Wald F statistic	24.573			
Overidentification test *p* value	0.0786			
Observations	1046	1046	1046	1046

Note: *** indicates *p* < 0.01 here.

**Table 5:** Results for different propensity score matching techniques.

Matching Method	Treatment Group	Control Group	ATT	T Value
Nearest neighbour matching (1:2)	6.131	5.880	0.251 **	2.23
Nearest neighbour matching (1:4)	6.131	5.898	0.233 **	2.18
Radius matching	6.131	5.863	0.268 **	2.52
Kernel matching	6.131	5.888	0.243 **	2.39
Local linear regression matching	6.131	5.866	0.265 **	2.07

Note: For nearest neighbour matching and radius matching, a radius of 0.01 is chosen. ** indicates *p* < 0.05.

**Table 6:** Mechanism analysis: Step (1).

First Step	
Explanatory Variable	*HDDS*
Smartphone use	0.248 *** (0.088)
Control variables	Y
Regional fixed effects	Y
Observations	1046
F statistic	9.830
R^2^	0.136

Note: To save space, the control variables and constant terms are not reported here. *** indicates *p* < 0.01.

**Table 7:** Mechanism analysis: Step (2).

Second Step		
Explanatory Variables	Income	Online Food Shopping
Smartphone use	0.232 ** (0.097)	0.060 *** (0.018)
Control variables	Y	Y
Regional fixed effects	Y	Y
Observations	1046	1046
F statistic	4.520	6.260
R^2^	0.067	0.119

Note: To save space, the control variables and constant terms are not reported here. *** indicates *p* < 0.01, ** indicates *p* < 0.05.

**Table 8:** Mechanism analysis: Step (3).

Third Step	
Explanatory Variable	*HDDS*
Smartphone use	0.201 ** (0.088)
Income	0.084 *** (0.028)
Online food shopping	0.388 *** (0.117)
Control variables	Y
Regional fixed effects	Y
Observations	1046
F statistics	10.610
R^2^	0.154

Note: To save space, the control variables and constant terms are not reported here. *** indicates *p* < 0.01, ** indicates *p* < 0.05.

**Table 9:** Heterogeneity analysis.

Explained Variable	Age		Gender		Income	
	(1)	(2)	(3)	(4)	(5)	(6)
	>45	≤45	Male	Female	>14,423.84	≤14,423.84
*HDDS*	0.230 ** (0.093)	0.158 (0.291)	0.336 *** (0.115)	0.152 (0.137)	0.393 *** (0.136)	0.091 (0.116)

Note: To save space, the control variables and constant terms are not reported here. *** indicates *p* < 0.01, ** indicates *p* < 0.05.

## Data Availability

The data presented in this study are available on request from the authors.
